# Echocardiography-guided percutaneous per-ventricular laser ablation of ventricular septum: in vivo study in a canine model

**DOI:** 10.1007/s10103-016-1881-3

**Published:** 2016-02-09

**Authors:** Guangbin He, Chao Sun, Xiangkong Zhang, Lei Zuo, Haiying Qin, Minjuan Zheng, Xiaodong Zhou, Liwen Liu

**Affiliations:** Ultrasound Department, Xijing Hospital, Fourth Military Medical University, Changle West Road 15 Hao, Xi’an, ShaanXi Province 710032 China; Ultrasound Department, Ningxia Medical University, Yin Chuan, China

**Keywords:** Laser ablation, Septum, Echocardiography, Percutaneous, Per-ventricular, Intervention

## Abstract

Surgical myectomy and ethanol ablation are established intervention strategies for left ventricular outflow obstruction in hypertrophic cardiomyopathy. Safety and efficacy limitations of these interventions call for a minimally invasive, potentially safer, and more efficacious strategy. In this study, we aimed to evaluate the feasibility of echocardiography-guided percutaneous per-ventricular laser ablation of a ventricular septum in a canine model. Six domestic dogs were chosen for the study. A 21G needle was inserted into the right ventricle with its tip reaching the targeted basal to mid-septum, after which laser ablation was performed as follows: 1-W laser for 3 min (180 J) at the basal segment and 5 min (300 J) at middle segment of the septum, respectively. Echocardiography, blood chemistry tests, and pathology examination were performed to assess the results of laser ablation. No death or major complications, i.e., tamponade, pericardial effusion, or ventricular fibrillation, occurred. The laser-ablated areas were well demarcated in the results of the pathological examination. The diameters of the ablated regions were 4.42 ± 0.57 and 5.28 ± 0.83 mm for 3 and 5 min ablation, respectively. Pre-ablation and post-ablation, cardiac enzymes were found to increase significantly while no significant differences were found among M-mode, 2D (LVEF), pulsed-wave (PW) Doppler, and tissue Doppler imaging (TDI) measurements. Contrast echocardiography confirmed the perfusion defects in the ablated regions. Microscopically, the ablated myocardium showed coagulative changes and a sparse distribution of disappearing nuclei and an increase in eosinophil number were observed. Our study suggests that percutaneous and per-ventricular laser ablation of the septum is feasible, potentially safe and efficacious, and warrants further investigation and validation.

## Introduction

Hypertrophic cardiomyopathy (HCM) is an autosomal dominant disease of the cardiac sarcomere with an occurrence of 1:500 worldwide [1]. HCM mainly manifests as left ventricular (LV) hypertrophy (≥15 mm) [2] and left ventricular outflow tract (LVOT) obstruction (accounting for up to 70 % of patients) [3], thereby leading to heart failure with dyspnea, chest pain, atrial fibrillation, and sudden death. Thus, most treatment strategies are directed at enlarging the LVOT and relieving the LVOT obstruction. Conservative medications (β-blockers, calcium-channel blockers, and anti-arrhythmic agents) are used to treat the vast majority of patients. Invasive therapy, which includes surgical myectomy, septal ethanol ablation, and dual-chamber pacing is introduced to patients with refractory symptoms or drug resistance. Surgical septal myectomy generally involves resection of hypertrophic tissue from the basal septum and has been advocated as the gold standard for symptomatic patients with severe complications [4]. Ethanol ablation is a less invasive approach that is performed by chemically inducing myocardial infarction and is an effective method for reducing septal thickness and relieving LVOT pressure gradients [5]. Studies have shown that both treatments improve functional status with similar HCM-related mortality [6], but no randomized trials comparing the two procedures have been performed [7]. Note that postoperative complications of left bundle branch block and right bundle branch block were found, and the occurrence rates were up to 46 and 40 % for surgical myectomy and ethanol ablation, respectively [8]. Considering the sternotomy and relatively high patients’ tolerance required in myectomy, the potentially risky misplacement of ethanol and the anatomic variability of the vascularized hypertrophic septum, and the potential risk of conduction block after these two treatments, the development of new minimally invasive approach is warranted.

Transcatheter intervention therapies, which include radio frequency (RF), microwave (WMA), laser, and cryoablation, have been extensively studied for the treatment of cardiac diseases, such as atrial fibrillation [9], ventricular tachycardia [10, 11], and pulmonary atresia [12]. Regarding septal ablation, the advantages of transcatheter RF ablation over ethanol ablation and myectomy include high accuracy and low risk of damaging the conduction system [13]. The mechanism of RF septal ablation to reduce LVOT gradient is by hypokinesis or akinesis of the ablated region [14], rather than thinning the septum in surgical septal myectomy [4]. High-intensity focused ultrasound (HIFU) was reported to be efficacious in ablating the septum and the LV free wall in canine myocardium without influencing the cardiac function [15]. However, given the difficulty in controlling the HIFU energy, the transducer size and the pulmonary vein isolation hamper the application of this technique. Laser has a primary advantage in magnetic resonance imaging (MRI) compatibility because it can be coupled with optical fiber [16]. It shows less efficiency in ablation compared with RF and WMA because of its scatter and rapid absorption by tissues [17]. However, this makes the progression of laser ablation more controllable as a precise and efficient light source for ablation.

From the mentioned studies, refinement in ablation technology facilitates the widespread application of this technique. Most minimal invasive ablation systems use a transcatheter during operation. Complications of transcatheter ablation, including thromboembolism, pulmonary vein stenosis [18], stroke, cardiac tamponade, and esophageal perforations, may occur [19]. Moreover, a long period of radiation exposure is unavoidable for both operator and patient during the transcatheter procedure. Thus, we aimed to investigate a potentially safer and more efficacious strategy for septum ablation. Minimal invasive percutaneous ablation has been widely used on focal benign gynecologic tumors [20] and on hepatic, renal, and lung malignancies [21, 22]. To date, few studies have been reported on percutaneous cardiac ablation without the use of a catheter. The occurrence of severe complications, such as pericardial tamponade, pericardial effusion, or ventricular fibrillation during the operation, is unclear. The variation in global and regional cardiac function during and after ablation also remains unknown.

The aim of this paper is to validate the feasibility of echocardiography-guided percutaneous per-ventricular laser ablation of the canine ventricular septum. An in vivo experiment was performed by using a laser inserted from the apical region on the right ventricle (RV) side to the targeted septum under the guidance of echocardiography. Echocardiography, blood chemistry tests, and pathology examination were performed to assess the results of laser ablation.

## Materials and methods

### Animal preparation

All animal experiments were approved by the local animal ethical committee for research animal care and performed in accordance with the ethical standards of the Declaration of Helsinki. Six domestic dogs, weighing 13 ± 2 kg, were anesthetized with an intravascular injection of 1.5 ml (0.1 ml/kg, as required) xylazine hydrochloride (Hua Mu Animal Care Inc., Ji Lin, China), followed by 0.75 ml of the same drug to maintain anesthesia. The hair around the heart region was removed before the operation. Intravascular injection of saline was given during the operation.

### Echocardiography-guided percutaneous laser ablation of in vivo canine ventricular septum

**Fig. 1:**
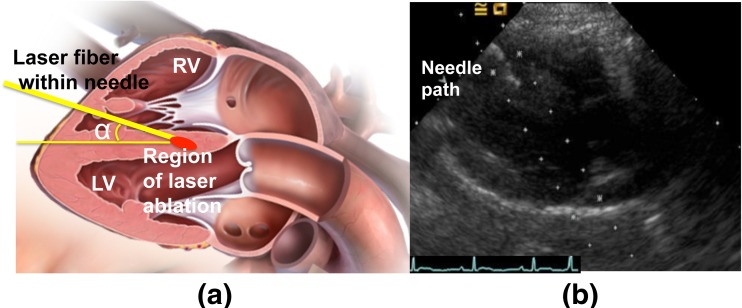
**a** Diagram of the needle path and ablation area ((http://medmovie.com/library_id/7556/) is acknowledged), **b** B-mode ultrasound image showing the needle path in parasternal LV long-axis view, *LV* left ventricle, *RV* right ventricle. The angle α of insertion against the septum in Fig. 1a maintains less than 45° in the process of the insertion and ablation

Neodymium-yttrium-aluminum-garnet laser (Nd:YAG, 800–1064 nm wavelength, 300-μm-diameter fiber, Echo Laser X4, Elesta S.R.L., Italy) was connected to an ultrasound scanner (MyLab 9.0, Esaote, Italy) for laser ablation, under the guidance of another ultrasound scanner (Acuson Sequoia 512, Siemens, Germany) with a transducer 4V1-C. In view of LV long axis, a needle (21G, PTC, ECOCHIBA, Italy) was injected into the RV obliquely from the apical area via percutaneous route, as shown in the diagram of Fig. 1a. The angle of insertion against the septum α in Fig. 1a was approximately less than 45°. The tip of the needle was inserted into the target septum along the guideline shown Fig. 1b. Note that the papillary muscle and chordae tendineae in RV were avoided under the guidance of the ultrasound during the insertion. Then, the laser fiber was inserted through the needle, and its contact with the septum was maintained. Each dog received two ablations. The first ablation was located at the basal segment of ventricular septum (avoiding the membranous segment) using a 1-W laser for 3 min (180 J). The second ablation was located at the middle segment using a 1-W laser for 5 min (300 J). Heart rate and blood pressure were recorded before and after the ablation.

### Echocardiographic evaluation

Two-dimensional echocardiography was acquired before ablation and immediately after ablation by applying an ultrasound scanner (iE33, Philips, The Netherlands) and transducer S5-1 (frequency: 1–5 MHz) to evaluate the variation in cardiac function. The measured echocardiographic parameters included the following: ejection fraction (EF), motion of the regional septum (at basal, middle, and apical segment) and posterior wall (basal, middle, and apical segments) in M-mode, ratio of peak early mitral inflow velocity (E-wave) and peak late mitral inflow velocity (A-wave) (E/A) in the pulsed-wave (PW) Doppler, E′/A′, measured at the mitral valve (MV) annulus, the peak systolic (Sa) and diastolic waves (Ea and Aa) in the velocity spectrum of tissue Doppler imaging (TDI) at the ablated area, and pressure gradient in LVOT. Each dataset was measured from three cardiac cycles.

### Serology examination

Blood (5 ml) was intravenously drawn from each dog before ablation and 3 hours after ablation. The cardiac enzymes, including aspartic acid transaminase (AST), lactate dehydrogenase (LDH), creatine kinase (CK), and creatine kinase isoform (CK-MB), were examined.

### Gross and pathological examinations

All dogs were sacrificed after collecting blood samples, and the hearts were removed for the gross and pathological examinations. Each heart was dissected to approximately 1-mm-thick slices to expose and carefully ascertain the range of ablation lesions. Maximal lesion length (D_1_), width (D_2_), and depth (D_3_) were measured, and the mean diameters (D_1_ + D_2_ + D_3_)/3 and mean volumes (0.5233 D_1_D_2_D_3_) [15] were calculated. Then, the ablated cardiac specimens were fixed in 10 % phosphate-buffered formalin (pH 7) and sent for histopathological examinations.

### Statistics

The results were presented as mean ± standard deviation. A paired *T* test (two-tailed) was performed using MATLAB software (MATLAB 2010a, The MathWorks Inc., Natick, MA, USA). The significance level was set at 0.05, and *P* values less than 0.05 indicated a statistically significant difference.

## Results

All dogs survived during and after the process of ablation without major physiological complications (e.g., pericardial tamponade, pericardial effusion, and ventricular fibrillation). No damage to papillary muscle and chordae tendineae in RV was found. Before and after ablation, the heart rates (91.7 ± 16.1 vs. 81.1 ± 29.6 bpm; *P* > 0.05) and blood systolic pressures (112.8 ± 8.1 vs. 116.4 ± 7.3 mmHg; *P* > 0.05) showed no significant difference.

### B-mode and contrast evaluation

**Fig. 2:**
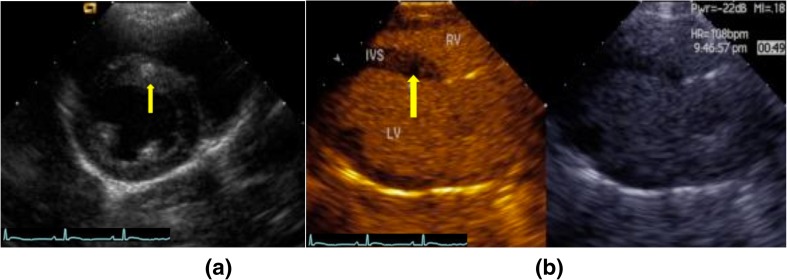
**a** A two-dimensional image showing the position of ablation (*arrows*) on the ventricular septum in LV short-axis view. Hyper-echo region indicates the ablated region. **b** Contrast mode in parasternal LV long-axis view, *LV* left ventricle, *RV* right ventricle, *IVS* interventional septum. Defect in blood filling is found at the ablation site in the *left panel* of Fig. 2b

The B-mode image in Fig. 2a shows the ablated regions in LV short-axis view. The hyper-echogenic area (arrow) is the ablated tissue appearing thicker than the surrounding tissues. In contrast mode (Fig. 2b), perfusion of the ultrasound contrast agents fills the LV and RV, thereby delineating the septum and posterior wall. No blood flow is found in the ablated region (yellow arrow) of the septum.

### Echocardiographic evaluation

**Fig. 3:**
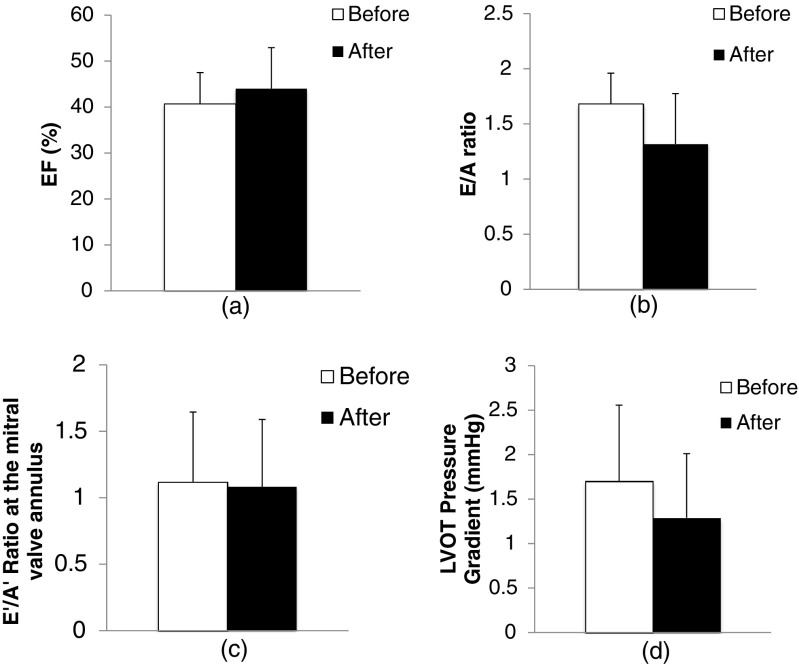
Variation in global systolic and diastolic functions pre- and post-ablation: **a** EF, **b** E/A ratio, **c** E′/A′ ratio at the MV annulus, **d** pressure gradient at LVOT

**Fig. 4:**
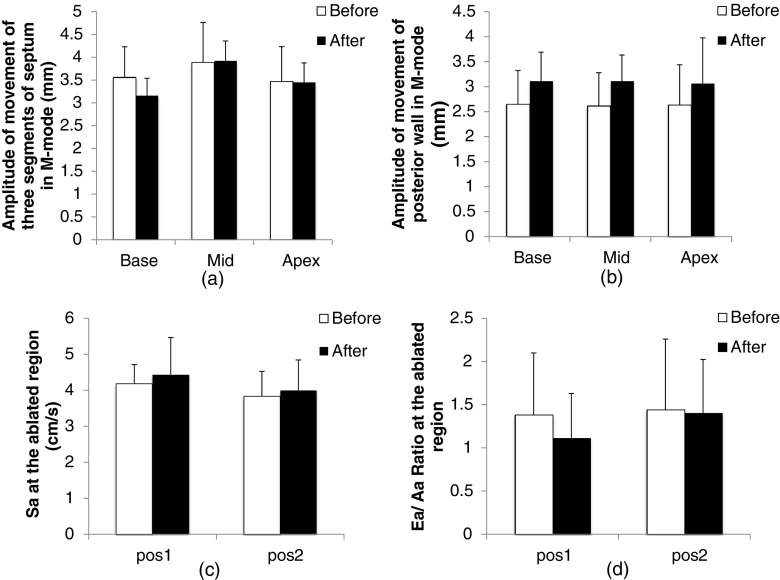
Variation in regional systolic and diastolic parameters pre- and post-ablation: **a** amplitude of movement of the basal, middle, and apical segments of the septum in M-mode; **b** amplitude of the movement of the basal, middle, and apical segments of the posterior wall in M-mode; **c** the peak systolic wave (Sa) in TDI at the ablated area; **d** Ea/Aa ratio at the ablated area

In Figs. 3 and 4, no significant differences are found in the global and regional systolic and diastolic functions before and after the ablation. Factors including EF (Fig. 3a), E/A ratio (Fig. 3b), E′/A′ ratio (Fig. 3c), pressure gradient of LVOT (Fig. 3d), amplitude of septal movement (Fig. 4a) and posterior wall (Fig. 4b) in M-mode, TDI velocity (Sa) (Fig. 4c), and Ea/Aa ratio at the ablated zone (Fig. 4d) are assessed.

### Serologic changes

**Fig. 5:**
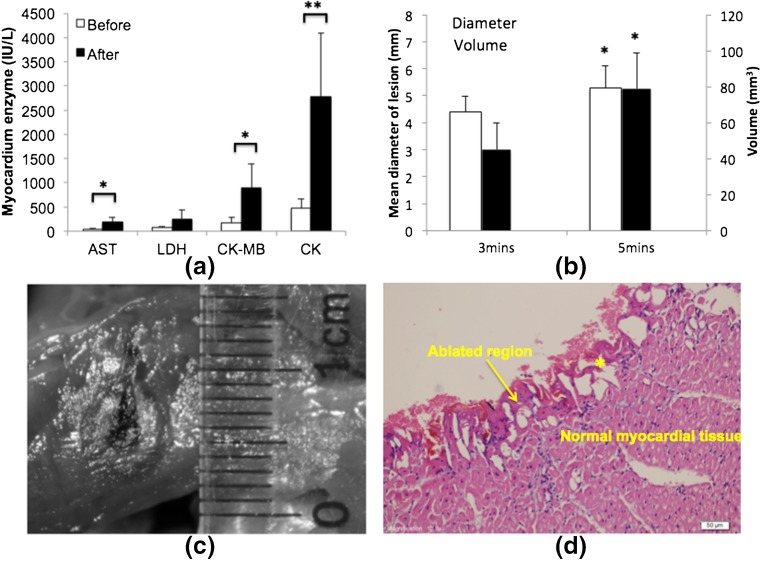
**a** The variation in cardiac enzyme before and after laser ablation (*P < 0.05, **P < 0.01); **b** the measured mean diameter and volume of the lesions; **c** example of the ablation zone, the center black region was charred and surrounded by white coagulated regions; and **d** pathological examinations of lesions showing the comparison of the ablated regions and the normal myocardial tissue. *Asterisk* shows the red blood cells infiltrating the widened intercellular space (hematoxylin and eosin ×200)

In Fig. 5a, cardiac enzymes (AST, CK-MB, and CK) significantly increase due to ablation of the myocardial cells. No significant increase in LDH is found before and after ablation. LDH is generally elevated on the second day after acute myocardial infarction and peaks on the fourth day [23]. Thus, serum test after 3 hours may be too short to detect the elevation in LDH.

### Gross and pathological examinations

The mean diameter and volume after 5 min ablation are significantly larger than the region ablated for 3 min in Fig. 5b. Figure 5c shows an example of myocardial lesions ablated for 5 min. The charred and coagulated necrotic regions are clearly seen with borders that distinguished these regions from the surrounding normal healthy myocardial tissue. A representative pathological examination of ablated myocardial lesion at ×200 magnification is shown in Fig. 5d. Normal myocytes are distributed evenly (arrow head) with the nuclei showing small dark-blue or purple precipitate. The ablated myocardial tissue (arrow head) is contracted, and the cells close to the laser show abnormal morphological characteristics. A zone of vacuoles forms a clear boundary between the ablated and normal tissues, and red blood cells infiltrate the widened intercellular space. Compared to normal tissue, the ultrastructure of the lesion is damaged and sparsely distributes, with fewer nuclei and an increasing number of eosinophils.

## Discussion

### The effectiveness of the echocardiography-guided laser ablation of the septum

Laser ablation of the septum is a feasible process, and no major physiological reactions or complications such as pericardial tamponade, pericardial effusion, and ventricular fibrillation were observed during and after ablation. Global and regional cardiac functions from echocardiographic parameters did not vary significantly before and after ablation. The basal segment of the septum presented a decreasing trend in amplitude of movement in M-mode pre-ablation compared with post-ablation, whereas the amplitude of movement of the three segments of posterior wall showed an increasing trend in M-mode. The increasing trend in amplitude of movement of the posterior wall indicated the compensatory action that maintained the systolic function. This observation agreed with the results obtained from the regional TDI hypokinesis of the ablated region without significant variation in global cardiac function [15]. Decreasing diastolic function (both global parameters of E/A, E′/A′, and regional parameter of Ea/Aa) was found post-ablation compared with pre-ablation. A decrease in diastolic function is known to be a sensitive indicator of cardiac dysfunction. This trend was possibly due to impaired myocardial relaxation which resulted in a decrease in the filling reliance [24].

The results of pathology and the increase of myocardial enzymes indicated damage to the myocardium. Tissue necrosis was clearly observed and suggested irreversible denaturation of proteins mainly due to thermal injury [25]. Around the ablation crater, visible whitening of irradiated tissue was observed, thereby indicating thermal coagulation as shown in Fig. 5c [26]. The pathological figure in Fig. 5d showed a zone of vacuoles lying beneath fragments of carbon in all lesions. Compared with other thermal ablation approaches, the laser required a longer time to create lesions. For example, the insonation time lasted a few seconds when HIFU ablation was performed at an output power of 2000–3000 W [15]. Trans-arterial argon laser myoplasty (wavelength 454–514 nm, power 2–2.2 W, exposure time 12–20 min, total energy 1440–2640 J) was reported, and the in vivo ablated region [27] was comparable with that observed in this study. In our study, the 1-W power Nd:YAG laser achieved the desired ablated region in a relatively short time (3–5 min) with a low intraoperative stimulus. The thickness of the canine septum was approximately 6 mm. Low-power laser instead of high power was utilized to obtain a controllable lesion size without rapid perforation [28]. The large charred region caused by high-power laser was expected to reduce the depth of laser penetration and caused possible damage to the optical fiber [29]. Hence, considering operational safety, the laser in this study was shown to be a safe method for in situ cardiac thermal ablation.

### Selection of the insertion path

The needle was inserted into the septum from the apical region on the RV side in this study. This was due to the lower pressure and slower blood flow in this area than from the LV route minimizing the occurrence of cardiac tamponade. Caution must be taken when controlling the insertion angle and depth of insertion. The needle was inserted obliquely along the septum because of the anatomical limitations of the canine chest and to reduce the risk of interventricular septum defect. Vertical insertion should be carefully avoided to minimize the influence of movement caused by respiration. A distance of 1.5–2 mm from the needle tip to the distal end of the septum on the LV side in the B-mode image would be appropriate.

### Limitation and prospects

This study focused on the short-term response of echocardiography-guided percutaneous laser ablation of healthy canine ventricular septum. Future work includes the long-term observation of safety of this approach on an animal model with hypertrophic myocardium and investigation of the underlying mechanism of septum reduction. Furthermore, no significant variation in cardiac function was found based on conventional echocardiographic parameters. Other sensitive parameters like strain may be applied to monitor the influence of laser ablation on the myocardium in animals of a larger scale. The standard deviation values in the echocardiographic results were relatively large because the incident angle of the needle was not completely identical for each animal. Under the guidance of echocardiography, we tried to insert the needle in the position that was closest to the initially targeted region. The integrity of the canine chest was well preserved, and the anatomy of the canine heart and the movement caused by respiration limited the reproducibility of needle insertion to some extent.

## Conclusion

This study investigated the feasibility of the echocardiography-guided percutaneous per-ventricular laser ablation on intraventricular septum of healthy canine. No major complications, such as pericardial tamponade, pericardial effusion, and ventricular fibrillation, occurred during and after ablation. Both pathological and serological results showed that using a 1-W laser for 3 and 5 min allowed the creation of lesions with effective coagulation and cardiac damage. Real-time echocardiography monitoring presented no significant variation in cardiac function during and after laser ablation. In summary, percutaneous and per-ventricular laser ablation of the septum is feasible, potentially safe and efficacious, and may become a viable alternative and a non-sternotomy solution to septum ablation. Long-term observation on the experimental outcomes in animals of HCM model warrants further investigation and validation.

## References

[1] Maron BJ, McKenna WJ, Danielson GK, Kappenberger LJ, Kuhn HJ, Seidman CE, Shah PM, Iii WHS, Spirito P, Cate FJT, Wigle ED, Vogel RA, Abrams J, Bates ER, Brodie BR, Danias PG, Gregoratos G, Hlatky MA, Hochman JS, Kaul S, Lichtenberg RC, Lindner JR, O’rourke RA, Pohost GM, Schofield RS, Tracy CM WLW, Klein WW, Priori SG, Alonso-Garcia A, Blomström-Lundqvist C, Backer GD, Deckers J, Flather M, Hradec J, Oto A, Parkhomenko A, Silber S, Torbicki A (2003). American College of Cardiology/European Society of Cardiology clinical expert consensus document on hypertrophic cardiomyopathy: a report of the American College of Cardiology Foundation Task Force on clinical expert consensus documents and the European Society of Cardiology Committee for Practice Guidelines. J Am Coll Cardiol.

[2] Enriquez AD, Goldman ME (2014). Management of hypertrophic cardiomyopathy. Ann Glob Health.

[3] Varma PK, Neema PK (2014). Hypertrophic cardiomyopathy: part 1—introduction, pathology and pathophysiology. Ann Card Anaesth.

[4] Olivotto I, Ommen SR, Maron MS, Cecchi F, Maron BJ (2007) Surgical myectomy versus alcohol septal ablation for obstructive hypertrophic cardiomyopathy: will there ever be a randomized trial? J Am Coll Cardiol 50(9):831–834. doi:10.1016/j.jacc.2007.05.018 10.1016/j.jacc.2007.05.018 17719467

[5] Rigopoulos A, Seggewiss H (2011). A decade of percutaneous septal ablation in hypertrophic cardiomyopathy. Circ J.

[6] Ball W, Ivanov J, Rakowski H, Wigle ED, Linghorne M, Ralph-Edwards A, Williams WG, Schwartz L, Guttman A, Woo A (2011). Long-term survival in patients with resting obstructive hypertrophic cardiomyopathy: comparison of conservative versus invasive treatment. J Am Coll Cardiol.

[7] Elliott PM, Anastasakis A, Borger MA, Borggrefe M, Cecchi F, Charron P, Hagege AA, Lafont A, Limongelli G, Mahrholdt H, McKenna WJ, Mogensen J, Nihoyannopoulos P, Nistri S, Pieper PG, Pieske B, Rapezzi C, Rutten FH, Tillmanns C, Watkins H (2014). 2014 ESC Guidelines on diagnosis and management of hypertrophic cardiomyopathy the Task Force for the Diagnosis and Management of Hypertrophic Cardiomyopathy of the European Society of Cardiology (ESC). Eur Heart J.

[8] Talreja DR, Nishimura RA, Edwards WD, Valeti US, Ommen SR, Tajik AJ, Dearani JA, Schaff HV, Holmes DR (2004). Alcohol septal ablation versus surgical septal myectomy—comparison of effects on atrioventricular conduction tissue. J Am Coll Cardiol.

[9] Doll N, Suwalski P, Aupperle H, Walther T, Borger MA, Schoon HA, Mohr FW (2008) Endocardial laser ablation for the treatment of atrial fibrillation in an acute sheep model. J Card Surg 198–203. doi:10.1111/j.1540-8191.2008.00601.x 10.1111/j.1540-8191.2008.00601.x 18435631

[10] Nitta T, Ikeshita M, Asano T, Tanaka S, Shoji T, S-i O, Sugiura M (1992). Surgical laser ablation of a pediatric idiopathic ventricular tachycardia. Ann Thorac Surg.

[11] Hirao K, Yamamoto N, Toshida N, Nawata H, Ishihara N, Suzuki F, Miyasaka N, Hiejima K, Tanaka M (1997). Transcatheter neodymium-yttrium-aluminum-garnet laser coagulation of canine ventricle using a balloon-tipped cardioscope. Jpn Circ J.

[12] Moskowitz WB, Titus JL, Topaz O (2004). Excimer laser ablation for valvular angioplasty in pulmonary atresia and intact ventricular septum. Lasers Surg Med.

[13] Lawren T, Borchert B, Leuner C, Bartelsmeier M, Reinhardt J, Strunk-Mueller C, Vilsendorf DMZ, Schloesser M, Beer G, Lieder F, Stellbrink C, Kuhn H (2011). Endocardial radiofrequency ablation for hypertrophic obstructive cardiomyopathy. J Am Coll Cardiol.

[14] Lucie R, Jan J, Josef V (2013). Ablation of hypertrophic septum using radiofrequency energy—an alternative for gradient reduction in patient with hypertrophic obstructive cardiomyopathy?. J Invasive Cardiol.

[15] Zheng M, Shentu W, Chen D, Sahn DJ, Zhou X (2014). High-intensity focused ultrasound ablation of myocardium in vivo and instantaneous biological response. Echocardiography.

[16] Stollberger R, Ascher PW, Huber D, Renhart W, Radner H, Ebner F (1998). Temperature monitoring of interstitial thermal tissue coagulation using MR phase images. J Magn Reson Imaging.

[17] Skinner MG, Iizuka MN, Kolios MC, Sherar MD (1998). A theoretical comparison of energy sources-microwave, ultrasound and laser-for interstitial thermal therapy. Phys Med Biol.

[18] Robbins IM, Colvin EV, Doyle TP, Kemp WE, Loyd JE, McMahon WS, Kay GN (1998). Pulmonary vein stenosis after catheter ablation of atrial fibrillation. Circulation.

[19] Doll N, Borger MA, Fabricius A, Stephan S, Gummert J, Mohr FW, Hauss J, Kottkamp H, Hindricks G (2003). Esophageal perforation during left atrial radiofrequency ablation: is the risk too high?. J Thorac Cardiovasc Surg.

[20] Carrafiello G, Recaldini C, Fontana F, Ghezzi F, Cuffari S, Lagana D, Fugazzola C (2010). Ultrasound-guided radiofrequency thermal ablation of uterine fibroids: medium-term follow-up. Cardiovasc Intervent Radiol.

[21] Ahmed M, Brace CL, Lee FT, Goldberg SN (2011). Principles of and advances in percutaneous ablation. Radiology.

[22] McTaggart RA, Dupuy DE (2007). Thermal ablation of lung tumors. Tech Vasc Interv Radiol.

[23] MacDonald RP, Simpson JR, Nossal E (1957). Serum lactic dehydrogenase—a diagnostic aid in myocardial infarction. J Am Med Assoc.

[24] Rasalingam R, Makan M, Perez JE (2012) The Washington manual of echocardiography. Lippincott Williams & Wilkins, Wolters Kluwer, Philadelphia, USA

[25] Anderson RR, Ross EV (1994). Laser-tissue interactions. Cutaneous laser surgery: the art and science of selective photothermolysis.

[26] Thomsen S (1991). Pathologic analysis of photothermal and photomechanical effects of laser-tissue interactions. Photochem Photobiol.

[27] Bonin JD, Lojeski EW, Ahron A, Isner JM, Clarke RH, Pandian NG, Donaldson RF, Salem DN, Konstam MA, Payne DD (1984). Laser myoplasty for hypertrophic cardiomyopathy: in vitro experience in human postmortem hearts and in vivo experience in a canine model (transarterial) and human patient (intraoperative). Am J Cardiol.

[28] Stafford RJ, Fuentes D, Elliott AA, Weinberg JS, Kamran A (2010). Laser-induced thermal therapy for tumor ablation. Crit Rev Biomed Eng.

[29] Fried NM, Lardo AC, Berger RD, Calkins H, Halperin HR (2000). Linear lesions in myocardium created by Nd:YAG laser using diffusing optical fibers: in vitro and in vivo results. Lasers Surg Med.

